# Frontal Brain Activity and Subjective Arousal During Emotional Picture Viewing in Nightmare Sufferers

**DOI:** 10.3389/fnins.2020.585574

**Published:** 2020-09-30

**Authors:** Michelle Carr, Richard Summers, Ceri Bradshaw, Courtney Newton, Leslie Ellis, Erin Johnston, Mark Blagrove

**Affiliations:** ^1^Department of Psychology, Swansea University, Swansea, United Kingdom; ^2^The International Focusing Institute, Nyack, NY, United States

**Keywords:** nightmares, arousal, differential susceptibility, diathesis-stress, emotion regulation, frontal activation

## Abstract

Nightmares are intensely negative dreams that awaken the dreamer. Frequent nightmares are thought to reflect an executive deficit in regulating arousal. Within a diathesis-stress framework, this arousal is specific to negative contexts, though a differential susceptibility framework predicts elevated arousal in response to both negative and positive contexts. The current study tested these predictions by assessing subjective arousal and changes in frontal oxyhemoglobin (oxyHB) concentrations during negative and positive picture-viewing in nightmare sufferers (NM) and control subjects (CTL). 27 NM and 27 CTL subjects aged 18–35 rated subjective arousal on a 1–9 scale following sequences of negative, neutral and positive images; changes in oxyHB were measured by Near-Infrared Spectroscopy (NIRS) using a 2 × 4 template on the frontal pole. Participants also completed the Highly Sensitive Person Scale, a trait marker for differential susceptibility; and completed a dream diary reporting negative and positive dream emotionality. The NM group had higher trait sensitivity, yet higher ratings of negative but not positive emotion in diary dreams. NM compared to CTL subjects reported higher subjective arousal in response to picture-viewing regardless of valence. Dysphoric dream distress, measured prospectively, was negatively associated with frontal activation when viewing negative pictures. Results suggest NM sufferers are highly sensitive to images regardless of valence according to subjective measures, and that there is a neural basis to level of trait and prospective nightmare distress. Future longitudinal or intervention studies should further explore positive emotion sensitivity and imagery in NM sufferers.

## Introduction

Nightmares are defined as intensely negative and unpleasant dreams that will awaken the dreamer, and are associated with distress upon awakening (Blagrove and Haywood, 2006; Robert and Zadra, 2014). Previous studies have shown around 2–6% of the population experience frequent, weekly nightmares (Hasler and Germain, 2009), which qualifies as having nightmare disorder (American Psychiatric Association, 2013).

According to the neurocognitive model, nightmares are thought to result from a deficit in executive function, particularly reflecting an executive deficit in regulating arousal (Levin and Nielsen, 2007, 2009). In support of this model, impaired executive functions have been found in those with frequent nightmares as shown by their performance in different neuropsychological tasks (Simor et al., 2012). In a replication study, those with frequent nightmares were found to have higher perseveration errors during a verbal fluency task (Carr et al., 2017), supporting the argument that specific frontal regulatory deficits are a neural correlate of nightmares. A recent finding that further complements this argument is that in individuals who experience frequent nightmares (≥2 bad dreams or nightmares per week), nightmare distress was shown to be associated with a decrease in frontal activity during a negative picture-viewing task (Marquis et al., 2019). The results suggest a possible overlap in brain mechanisms that are involved in nightmare dysphoria (during sleep) and distress (during wake) among those who frequently recall nightmares.

The neurocognitive model of nightmares fits within a diathesis-stress framework in that it argues that individuals high in trait affect distress are more vulnerable to waking stress and develop frequent, distressful nightmares in response (Levin and Nielsen, 2007, 2009). At a trait level, affect distress bears similarity to several negative personality traits, such as hyperarousal and neuroticism (Blagrove et al., 2004); affect distress is also thought to develop in response to a history of adverse experiences or trauma.

However, diathesis-stress frameworks limit focus to negative symptoms and outcomes, whereas the possible positive traits of nightmare-prone individuals are relatively unstudied. It was recently proposed that at a trait level, individuals who are prone to nightmares may instead be characterized by a general increase in sensitivity, and that this sensitivity may be associated with both nightmares and positive emotional experiences (Carr and Nielsen, 2017). This new model places nightmares within a differential susceptibility framework, which proposes that individual differences emerge not only in response to negative contexts, but also in response to positive or supportive contexts, as an increased sensitivity to the environment in general (Belsky et al., 2007; Belsky and Pluess, 2009). Specifically, the trait of ‘sensory-processing sensitivity’ describes an increase in emotional reactivity, greater depth of processing, and subtle awareness of environmental stimuli (Aron and Aron, 1997). Sensory-processing sensitivity bears similarity to another trait known to correlate with nightmare frequency—boundary thinness. Both traits are associated with general increased sensitivity, which is evident in dreams and waking experiences (Hartmann, 1984). Both have been associated not only with nightmares but also with vivid daydreams and dreams.

The trait of sensory-processing sensitivity has been studied in a range of contexts; at a neural level, highly sensitive individuals show increased responsiveness to both negative and positive images (Acevedo et al., 2017), to visual scenes (Jagiellowicz et al., 2011), and to social stimuli (Aron and Aron, 1997). Sensory-processing sensitivity functions within a differentially susceptible manner: highly sensitive individuals are more likely to experience distress and psychopathology in response to stressors, yet also process positive experiences more deeply, which can be associated with adaptive outcomes (Greven et al., 2019). Applied to the study of nightmares, a differential susceptibility framework would predict that nightmare-prone individuals are highly sensitive, and that while they experience greater sensitivity to negative contexts and increased nightmare distress, they also experience greater sensitivity to positive contexts and increased positive imagery.

The current study aimed to contrast predictions of diathesis-stress and differential susceptibility frameworks by assessing nightmare sufferers on a range of questionnaire, dream diary, task and neuroimaging measures. A diathesis-stress framework would predict only negative traits and attributes be higher in nightmare sufferers, whereas differential susceptibility would predict higher negative and positive attributes. The current study expanded on Marquis et al. (2019) to assess changes in frontal oxyhemoglobin (oxyHB) concentrations during a negative, positive and neutral picture-viewing task using Near-Infrared Spectroscopy (NIRS). Overall, the study aimed to assess frontal brain activity and subjective arousal during an emotional picture-viewing task, collect sleep and dream diary reports at home, and measure general dream and emotional experiences through questionnaires. The main predictions of the study included that: (1) nightmare sufferers would have higher trait sensory-processing sensitivity than control subjects as measured by the Highly Sensitive Person Scale (Aron and Aron, 1997); (2) nightmare sufferers would have greater negative and positive dream imagery than control subjects; (3) nightmare sufferers would exhibit increased arousal in response to both negative and positive images compared to control subjects; (4) within nightmare participants, nightmare distress will negatively correlate with frontal activation during negative picture viewing (replicating Marquis et al., 2019) and (5) negatively correlate during positive picture-viewing according to a differential susceptibility, but not a diathesis-stress framework.

## Methods

### Participants

Data for this study were collected as part of a larger intervention study, and only baseline measures of the full dataset are included here (no other findings have been published as yet). Participants were recruited through the use of a University-wide email advertisement, where a different advertisement was posted targeting nightmare and control participants. All participants signed Informed Consent (ethics approved by the Department of Psychology Research Ethics Review).

All potential candidates were required to complete a set of screening questionnaires to assess their eligibility. Candidates were excluded if they exceeded clinical thresholds on the Beck Depression Inventory-II (Beck et al., 1996) or the Posttraumatic Stress Disorder Checklist (Civilian Version; as cited in Ruggiero et al., 2003). The screening questionnaire also included questions regarding the candidates’ retrospective dream, bad dream and nightmare recall frequencies per month, and ratings of nightmare distress and bad dream distress on a 1–9 Likert scale. For eligibility, those recruited had to recall either at least one nightmare or two bad dreams per week, or no more than one nightmare per month.

Fifty-five eligible participants were enrolled in the study and the final sample consisted of 54 participants (1 excluded for lack of response to dream diary and questionnaires). The mean age of the sample was 23.91 (SD = 4.20, range = 20). The gender distribution was 35 females (64.81%), and 19 males (35.19%).

Participants enrolled in the study first completed a measure of nightmare frequency and distress at intake (the Van Dream Anxiety Scale; Aǧargün et al., 1999). The Van Dream Anxiety scale assesses dream anxiety during the preceding month, with 13 self-rated questions regarding nightmares, including frequency, difficulty in falling asleep after a nightmare, and frequency of each of the following because of nightmares: fear of sleeping, trouble sleeping, anxiety, occupational/familial/social/psychological distress, and memory/concentration problems. Scores on this scale were used to median split the participants into a nightmare (NM) and control (CTL) group, as this measure was more current to participant intake, and encompassed both nightmare frequency and distress. In order to create the split, those scoring eight or more were placed into the NM group (*n* = 27; 17 female, 10 male; age = 23.63 ± 4.28), and those scoring seven and below made up the CTL group (*n* = 27; 18 female, 9 male; age = 23.77 ± 4.97). Van Dream Anxiety scores for the two groups were: NM = 15.22 ± 7.83; CTL = 2.44 ± 2.81, *t* = 7.98, *p* < 0.005. For the conceptual replication of Marquis et al. (2019), we conducted correlations within a subset of nightmare participants (*n* = 18) reporting a frequency of at least eight bad dreams or nightmares per month (comparable to at least two dysphoric dreams per week in Marquis et al., 2019).

### Measures

#### NIRS

The Near-Infrared Spectroscopy data were collected using the Artinis Medical Systems Oxymon-MKIII along with the proprietary Oxysoft software, which records and measures changes in frontal cortical blood flow during the task. In NIRS, near-infrared light is transmitted via optode transmitters, through the skull and outer cortical regions. The scattered light is then received through another optode, thus non-invasively measuring changes in raw optical density. Measurements were sampled at 10 Hz and by using the Modified Beer–Lambert law (Cope and Delpy, 1988), raw optical densities were converted into oxygenated and deoxygenated hemoglobin changes, which is taken to be an indirect indication of cortical activation. We used a 2 × 4 frontal optode holder with four transmitters and four receivers placed on the frontal pole. The locations of the optode co-ordinates were estimated using Polhemus Patriot, using 3D Extension. The average MNI co-ordinates were used and exported for channel registration (see Supplementary Table S1).

Near-Infrared Spectroscopy analysis was conducted using NIRS-SPM. The model was specified in seconds (task vs rest) 32 s on and 20 s off. The wavelet-minimum description length (wavelet-MDL) was used to remove an unknown global trend (noise) from breathing, cardiac, vaso-motion or other experimental errors. A low pass filter “HRF” was used to attenuate for high-frequency noise. A precoloring method was used to estimate temporal correlation of the NIRS data (Worsley and Friston, 1995). The General Linear Model (GLM) parameters and temporal correlations were estimated in NIRS-SPM and the Beta values generated were used in the second level of analysis. Beta values were generated for each channel, for each condition (negative, neutral, positive), and were subsequently averaged across the four channels in the left and right hemisphere.

#### Task

The task consisted of rating subjective arousal following sequences of negative, neutral, and positive pictures. The images were taken from the Nencki Affective Picture System (NAPS) database of emotional imagery (Marchewka et al., 2013). Four sets of 60 images (20 negative, 20 neutral, 20 positive) were developed in an earlier study, and the average valence and arousal ratings of the negative, neutral, and positive images were closely matched between all four sets based on the NAPS database ratings (Reid et al., 2018).

In the current study, participants were instructed to observe a series of four images at a time and then judge their level of arousal on a scale of 1–9 (1 = not at all aroused, 9 = highly aroused); participants also rated the emotional valence of the images (1 = negative, 5 = neutral, 9 = positive; these ratings were used to confirm image valence and are not further analyzed here). PsychoPy was used to display the images, as well as record participants’ arousal and valence responses. Each image was presented for 8 s each, for a total of 32 s per trial. Participants then completed the arousal and valence scales, followed by a 20-s rest period. The images were presented in blocks of neutral, negative, and positive images (randomized block order). Within each valence block, there were five categories of images, including four images each of objects, landscapes, animals, people, and faces (randomized category order).

#### Questionnaires

The Warwick-Edinburgh Mental Well-being Scale (Stewart-Brown and Janmohamed, 2008). This scale monitors the mental well-being of the general population. It is a 14-item scale with five response categories, summed to provide a single score ranging from 14 to 70. The items are all worded positively and cover both feeling and functioning aspects of mental well-being.

The Dream Lucidity Questionnaire (DLQ; Stumbrys et al., 2013). The DLQ measures different aspects of lucidity within dreams. It consists of 14 items, scored on a 5-point scale, that evaluate different types of awareness, control and memory in dreams.

The Mannheim Dream Questionnaire (MADRE; Schredl et al., 2014). The MADRE questionnaire includes 21-items regarding the frequency of recalling dreams, nightmares, and lucid dreams, as well as questions about attitude toward dreams; we specifically analyzed the subscale of attitude toward dreams here (ATD).

The Interpersonal Reactivity Index (IRI; Davis, 1980). The IRI is a 28-item questionnaire that assesses subjective reactions to the observed experiences of another person, answered on a 5-point Likert scale. The measure has four subscales, including Perspective Taking, Fantasy, Empathic Concern, and Personal Distress.

The Highly Sensitive Person Scale (HSPS; Aron and Aron, 1997). The HSPS assesses sensory-processing sensitivity on a 27-item scale. Items on this scale range from being more sensitive to pain, hunger, and caffeine, to being deeply moved by the arts and music or startling easily.

#### Dream Diary

Each morning participants filled in a brief dream diary, answering questions regarding their sleep and dream quality in the previous night. Participants completed between 4 and 8 weeks of dream diaries in the intervention study but we here analyzed only the first week as a baseline measure of at-home dream quality. The diary was completed online via PsyToolkit. Participants log in with their unique ID, and are asked to report: time to bed, minutes to fall asleep, minutes awake during the night, and time out of bed (not analyzed here). Participants then completed a dream report followed by six scaled Likert questions (1–9 scale) regarding the intensity of negative and positive emotional content, body sensations, and waking impact of the dream. An option of “no recall” was also possible.

#### Analysis

Between group *t*-tests were conducted for all questionnaire measures. Between group ANOVAs were conducted for diary measures, including separate 2 group × 2 repeated measures ANOVAs for dream emotion intensity (negative, positive), dream body sensation intensity (negative, positive) and impact of the dream on waking mood (negative, positive). Between-group comparisons were conducted to assess differences in subject arousal in response to negative, neutral and positive picture-viewing using a 2 group × 3 repeated measures ANOVA. Within the subset of frequent nightmare participants, correlations were conducted between frontal NIRS beta values and retrospective dysphoric dream recall frequency for the past month, retrospective nightmare distress (score on Van Dream Anxiety Scale), and prospective dysphoric dream distress (diary measure of negative impact of dream on waking mood).

## Results

### Questionnaire Measures

Independent *t*-tests were conducted in order to compare the NM and CTL group on all questionnaire measures (see Table 1). NM subjects scored higher on the Highly Sensitive Person Scale (*p* < 0.005) while CTL subjects had higher well-being scores (*p* < 0.005). In addition to this, the personal distress and empathy subscales of the IRI trended toward being higher in the NM group (*p* < 0.07), although because eight *t*-tests were conducted, a corrected Bonferroni significance level would require *p* < 0.006.

**TABLE 1:** Means, standard deviation, and *p*-values for *t*-tests comparing NM and CTL groups on questionnaire measures.

****	**NM**	**CTL**		
	**Mean ± SD**	**Mean ± SD**	***T***	***p***
Highly Sensitive	4.82 ± 0.90	3.76 ± 0.91	–4.19	< 0.005*
Perspective taking	19.08 ± 3.43	18.65 ± 5.12	–0.87	0.73
Fantasy	19.42 ± 4.58	17.96 ± 7.26	–1.83	0.39
Empathy	21.73 ± 4.29	19.38 ± 4.94	–1.85	0.07
Personal distress	11.23 ± 4.18	9.31 ± 3.27	–1.89	0.07
Well-being	50.15 ± 10.84	59.22 ± 5.85	3.83	< 0.005*
Dream lucidity	29.63 ± 10.48	25.04 ± 11.53	–1.53	0.13
Attitude to dreams	39.56 ± 8.67	36.07 ± 8.18	–1.52	0.14

Corrected significance level of *p* < 0.006*.

### Dream Diary Measures

Three, 2 group (NM, CTL) × 2 valence (negative, positive) ANOVAs were conducted on emotion, sensation, and impact of the dream upon awakening. For emotion: The main effect of group was significant: *F*(1,52) = 5.30, *p* = 0.025, and there was a significant valence by group interaction: *F*(1,52), *p* < 0.0001. For sensation: The main effect of group was significant: *F*(1,52) = 10.92, *p* = 0.002, and there was a significant valence by group interaction: *F*(1,52), *p* < 0.0001. For impact: the main effect of group was significant: *F*(1,52) = 5.50, *p* = 0.023, and there was a significant valence by group interaction: *F*(1,52), *p* < 0.002. For the three dream attributes (emotion, sensation, impact), the NM group had higher ratings than the CTL group (group effect), and moreso for negative than positive ratings (valence by group interaction; see Table 2). Independent contrasts revealed only negative, not positive, ratings were significantly higher in NM compared to CTL group (*p* < 0.05).

**TABLE 2:** Mean and standard deviation scores of negative dream emotion, sensation and impact, and positive dream emotion, sensation and impact.

	**NM**	**CTL**

	**Mean ± SD**	**Mean ± SD**
Negative emotion	3.78 ± 1.38	2.26 ± 0.90
Negative sensations	3.33 ± 1.41	1.94 ± 0.80
Negative impact	3.16 ± 1.28	1.94 ± 0.72
Positive emotion	2.73 ± 1.16	2.59 ± 1.23
Positive sensations	2.27 ± 1.05	2.04 ± 1.01
Positive impact	2.97 ± 1.41	3.00 ± 1.60

### Task Subjective Arousal

**FIGURE 1:**
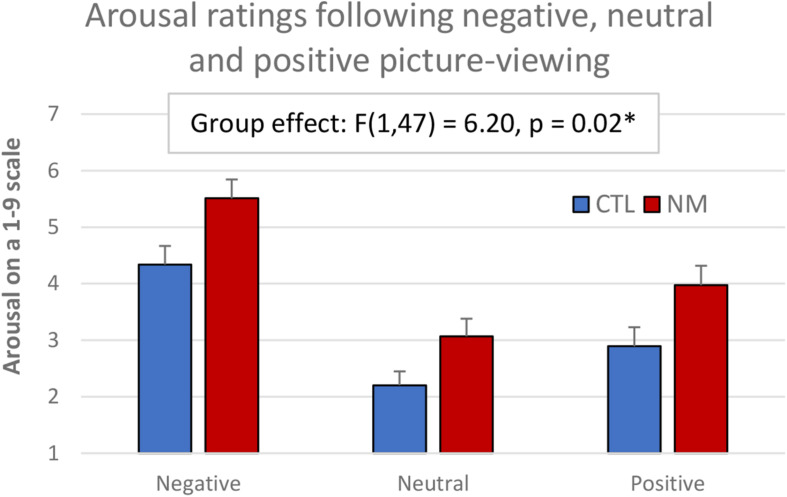
Subjective arousal ratings in NM and CTL participants following emotional picture-viewing.

A 2-group × 3-valence ANOVA was conducted on subjective arousal in response to the negative, neutral, and positive images (see Figure 1). The ANOVA showed a significant main group effect: *F*(1,50) = 6.51, *p* = 0.01, in that the NM group had higher subjective arousal than CTL group; and a main valence effect: *F*(2,100) = 71.23, *p* < 0.005, in that arousal was higher in negative, then positive, then neutral conditions (see Figure 1). There was no significant valence by group interaction: *F*(2,100) = 0.45, *p* = 0.64.

### Conceptual Replication of Marquis et al. (2019)

18 participants met the cut-off of having at least eight bad dreams or nightmares per month (comparable to at least two dysphoric dreams per week in Marquis et al., 2019). Marquis et al. (2019) conducted correlations between rCBF and retrospective dysphoric dream recall frequency (screening question per week), retrospective nightmare distress (measured by the Nightmare Distress Questionnaire, Belicki, 1992), and prospective dysphoric dream recall frequency (daily diary measure of frequency of dysphoric dreams). We here conducted correlations between NIRS beta values and retrospective dysphoric dream recall frequency (screening question per month), nightmare distress (measured by the Van Dream Anxiety Scale), and prospective dysphoric dream distress (using average daily diary rating of negative waking impact of a dream).

This set of analyses revealed negative correlations between nightmare distress and frontal activation during neutral picture-viewing (significant for left hemisphere, *p* < 0.05; trend on right, *p* = 0.08); and negative correlations between prospective dysphoric dream distress and frontal activation during negative picture viewing (significant on the right, *p* = 0.04; trend on the left, *p* = 0.08; see Table 3). No significant correlations were found between frontal activation and dysphoric dream measures in the subset of remaining (low nightmare frequency) participants.

**TABLE 3:** Analyses in subset of participants (*n* = 18) recalling at least 8 bad dreams or nightmares per month.

	**Dysphoric dream frequency (retrospective)**		**Nightmare distress (retrospective)**		**Dysphoric dream distress (prospective)**	
	***R***	***p***	***R***	***p***	***R***	***p***
LH-Negative	–0.182	0.47	0.025	0.92	–0.427	0.08^†^
RH-Negative	–0.039	0.88	–0.210	0.40	–0.489	0.04*
LH-Neutral	0.265	0.29	–0.472	< 0.05*	–0.166	0.51
RH-Neutral	0.230	0.36	–0.427	0.08^†^	–0.111	0.66
LH-Positive	0.179	0.48	–0.043	0.86	–0.206	0.41
RH-Positive	0.061	0.81	0.306	0.22	0.078	0.76

Correlations conducted between NIRS beta values and retrospective dysphoric dream recall frequency (# bad dreams and nightmares per month), retrospective nightmare distress (scores on Van Dream Anxiety Scale), and prospective dysphoric dream distress (average rating for negative waking impact of a dream on a 1–9 scale over a 1-week dream diary). *p < 0.05; ^†^*p* = 0.08.

## Discussion

The aim of the present study was to explore whether individuals with frequent nightmares are more highly sensitive and experience more negative and positive dream imagery, and more negative and positive emotional arousal in response to images. Regarding our main predictions, we found that: (1) nightmare sufferers have higher trait sensory-processing sensitivity than control subjects as measured by the Highly Sensitive Person Scale (Aron and Aron, 1997) as predicted; (2) nightmare sufferers have greater negative dream imagery than control subjects as predicted, but not positive imagery contrary to our prediction; (3) nightmare sufferers exhibit increased arousal in response to both negative and positive images compared to control subjects as predicted; (4) within nightmare participants, dysphoric dream distress is negatively correlated with frontal activation during negative picture viewing as predicted (replicating Marquis et al., 2019) and (5) contrary to predictions, dysphoric dream distress did not negatively correlate with frontal activation during positive picture-viewing.

Our questionnaire measures showed that in fact nightmare participants scored higher on the Highly Sensitive Person Scale. This is in line with a prior finding that nightmare sufferers are more highly sensitive than control subjects (Carr et al., 2020), providing further support to the notion that sensory-processing sensitivity is a trait marker for nightmares.

Analysis of the dream diary ratings showed that nightmare sufferers exhibit significantly more negative emotional intensity, negative body sensations, and distress from their dreams than did control subjects, although the two groups did not differ in the amount of positive emotion, sensations or impact of their dreams. While the finding does not provide evidence for differential susceptibility, it is known that patterns of negative or positive dream imagery change over time in response to waking stress. For instance, periods of high stress are associated with more nightmares, and resolution of recurrent nightmares is associated with greater well-being (Zadra et al., 1998). Thus a further test of differential susceptibility would require following patterns of dream imagery over time to see if nightmare-prone individuals might also experience intensified positive imagery during low-stress periods. Nevertheless, the current study suggests that at least during times marked by dysphoric dreaming, nightmare-prone individuals do not exhibit more positive dream imagery than control subjects.

During the picture-viewing task, we found that nightmare subjects rated their subjective level of arousal as significantly higher in response to both negative and positive images, as well as neutral images. This meets our prediction that nightmare sufferers have increased positive emotional arousal in addition to the typically studied negative emotional arousal, fitting within the framework of differential susceptibility. Nevertheless, our NIRS analysis did not follow predictions of differential susceptibility, and rather seemed more consistent with a diathesis-stress framework. Specifically, within nightmare participants, prospective dream distress negatively correlated with frontal activation during negative picture viewing; retrospective nightmare distress negatively correlated with frontal activation during neutral picture viewing. Our findings align with those of Marquis et al. (2019), who found measures of dysphoric dream recall and nightmare distress negatively correlated with cerebral blood flow during both a neutral and a negative picture-viewing task. On a related note, this provides some support for the idea that lucid dreams can be used to treat frequent nightmares, since lucid dreams seem to be characterized by an increase in frontal activity (Macêdo et al., 2019). That we did not see any correlations for the positive condition does not align with predictions of the differential susceptibility framework.

The use of NIRS does carry some limitations. While NIRS has good temporal resolution, it lacks specific spatial resolution due to relying on a BOLD response, and is subject to a hemodynamic lag. Because of this, NIRS tasks require the use of block designs, where repeated task exposure is coupled with periods of rest, to observe changes in blood flow at each task onset period compared to rest. In addition to this, NIRS is only able to measure ∼2 cm thickness of cortex, with added individual variables such as skull and hair thickness affecting the signal quality and depth. It is therefore reasonable to question whether the frontal activity measured in the current study adequately assessed executive frontal activation in response to the stimuli. As an exploratory conceptual replication of Marquis et al. (2019) we did not correct for multiple correlations, given that measures of frontal activation in different channels are not independent (Streiner, 2015); nevertheless the small sample size and lack of correction is a limitation of the current work warranting further investigation.

Overall, the study provides mixed support for the predictions of a differential susceptibility framework for nightmares. Nightmare sufferers are characterized by higher sensory-processing sensitivity, but exhibit predominantly negative dream imagery at least during the measured study period which, by definition, was marked by nightmares. Nightmare sufferers subjectively report higher arousal in response to both negative and positive, and neutral images, although the predicted negative correlations between nightmare distress and frontal activation were found only during negative and neutral picture viewing, in those with frequent nightmares. We suggest that the appropriate next step would be a longitudinal or cross sectional study to more adequately assess how variations in waking stress correspond with dream imagery in nightmare-prone, highly sensitive individuals. Further neuroimaging research may likewise bolster or refute our suggestion of bi-valent neural deactivation in nightmare-prone individuals.

## Data Availability Statement

The raw data supporting the conclusions of this article will be made available by the authors, without undue reservation.

## Ethics Statement

The studies involving human participants were reviewed and approved by Swansea University Department of Psychology Research Ethics Review. The participants provided their written informed consent to participate in this study.

## Author Contributions

MC: funding acquisition, conceptualization, data collection, data analysis, and manuscript writing and revision. RS, EJ, and CN: data collection, data analysis, and manuscript writing and revision. CB: data analysis, manuscript writing and revision, and supervision. LE: conceptualization, manuscript writing and revision. MB: funding acquisition, conceptualization, manuscript writing and revision, and supervision. All authors contributed to the article and approved the submitted version.

## Conflict of Interest

The authors declare that the research was conducted in the absence of any commercial or financial relationships that could be construed as a potential conflict of interest.

## Supplementary Material

Supplementary Table 1 Locations of NIRS channels.
