# Supplementary material for: Frontal Brain Activity and Subjective Arousal During Emotional Picture Viewing in Nightmare Sufferers
Source: Front Neurosci. 2020 Sep 30;14:585574. doi: 10.3389/fnins.2020.585574 (PMC7561419; doi:10.3389/fnins.2020.585574)
Supplement: Supplementary Table 1 — Locations of NIRS channels. [file Table_1.pdf]

1 Supplementary table

	Ch1	Ch2	Ch3	Ch4	Ch5	Ch6	Ch7	Ch8
x	45.33	34.00	55.33	45.00	-18.33	-31.67	-33.33	-46.33
y	58.67	64.33	40.67	47.67	70.33	55.33	66.00	50.67
z	-2.33	15.33	10.33	28.00	17.00	29.67	-2.33	11.33
Locations in Harvard-Oxford Cortical Structural Atlas	14% Frontal Pole	19% Frontal Pole	31% Frontal Pole	6% Frontal Pole	32% Frontal Pole	29% Frontal Pole	27% Frontal Pole	42% Frontal Pole

Table S1. *Locations of NIRS channels.*
